# Protection from Endotoxin Shock by Selective Targeting of Proinflammatory Signaling to the Nucleus Mediated by Importin Alpha 5

**DOI:** 10.4049/immunohorizons.1900064

**Published:** 2019-09-18

**Authors:** Yan Liu, Ruth Ann Veach, Jozef Zienkiewicz, Kelli L. Boyd, Taylor E. Smith, Zhi-Qi Xu, Lukasz S. Wylezinski, Jacek Hawiger

**Affiliations:** * Division of Allergy, Pulmonary and Critical Care Medicine, Department of Medicine, Vanderbilt University School of Medicine, Nashville, TN 37232; † Department of Veterans Affairs, Tennessee Valley Health Care System, Nashville, TN 37212; ‡ Division of Nephrology, Department of Medicine, Vanderbilt University School of Medicine, Nashville, TN 37232; § Department of Pathology, Microbiology and Immunology, Vanderbilt University School of Medicine, Nashville, TN 37232; ¶ Department of Molecular Physiology and Biophysics, Vanderbilt University School of Medicine, Nashville, TN 37232

## Abstract

Endotoxin shock is induced by LPS, one of the most potent virulence factors of the Gram-negative bacteria that cause sepsis. It remains unknown if either proinflammatory stress-responsive transcription factors (SRTFs), ferried to nucleus by importin *α*5, or lipid-regulating sterol regulatory element binding proteins (SREBPs), transported to the nucleus by importin β1, mediate endotoxin shock. A novel cell-penetrating peptide targeting importin *α*5 while sparing importin β1 protected 80% of animals from death in response to a high dose of LPS. This peptide suppresses inflammatory mediators, liver glycogen depletion, endothelial injury, neutrophil trafficking, and apoptosis caused by LPS. In d-galactosamine-pretreated mice challenged by 700-times lower dose of LPS, rapid death through massive apoptosis and hemorrhagic necrosis of the liver was also averted by the importin *α*5–selective peptide. Thus, using a new tool for selective suppression of nuclear transport, we demonstrate that SRTFs, rather than SREBPs, mediate endotoxin shock.

## INTRODUCTION

Humans mount a systemic inflammatory response to as little as 2–5 ng of LPS per kilogram of body weight. This reaction evokes a 2-fold increase in the expression of 4533 genes in circulating leukocytes, a transcriptional response known as a “genomic storm.” LPS is one of the most proinflammatory virulence factors of Gram-negative bacteria (1, 2). These multidrug resistant pathogens typically cause lung infections, which often leads to sepsis and septic shock, especially in immunocompromised hosts (2). LPS recognition by its cognate receptor, TLR4, expressed on multiple cell types (including myeloid and lymphoid cells, vascular endothelial cells, and respiratory epithelial cells), triggers robust signaling to the nucleus mediated by a cascade of signal transducers (3, 4). This signaling cascade unmasks the nuclear localization sequence (NLS) on NF-κB family members and other SRTFs (e.g., STAT1). Exposing these “zip codes” for nuclear import enables instant recognition by the nuclear transport shuttles, importin *α* (Imp *α*) and Imp β, thereby allowing nuclear translocation of SRTFs (5, 6). Thus, the nuclear transport system comprises the pivotal checkpoint for several transcriptional cascades (2).

Upon reaching the genome, SRTFs activate a myriad of genes that encode mediators of inflammation, such as cytokines, chemokines, cell-adhesion molecules (e.g., VCAM-1), and other intracellular signal transducers (2, 5). We have shown previously that this inflammatory genomic storm can be calmed by cell-penetrating peptides known as nuclear transport modifiers (NTMs), which target nuclear transport shuttles (7, 8). The NTM cSN50.1 was remarkably effective in LPS-induced inflammatory models (7) and a model of polymicrobial sepsis (6).

**FIGURE 1.**
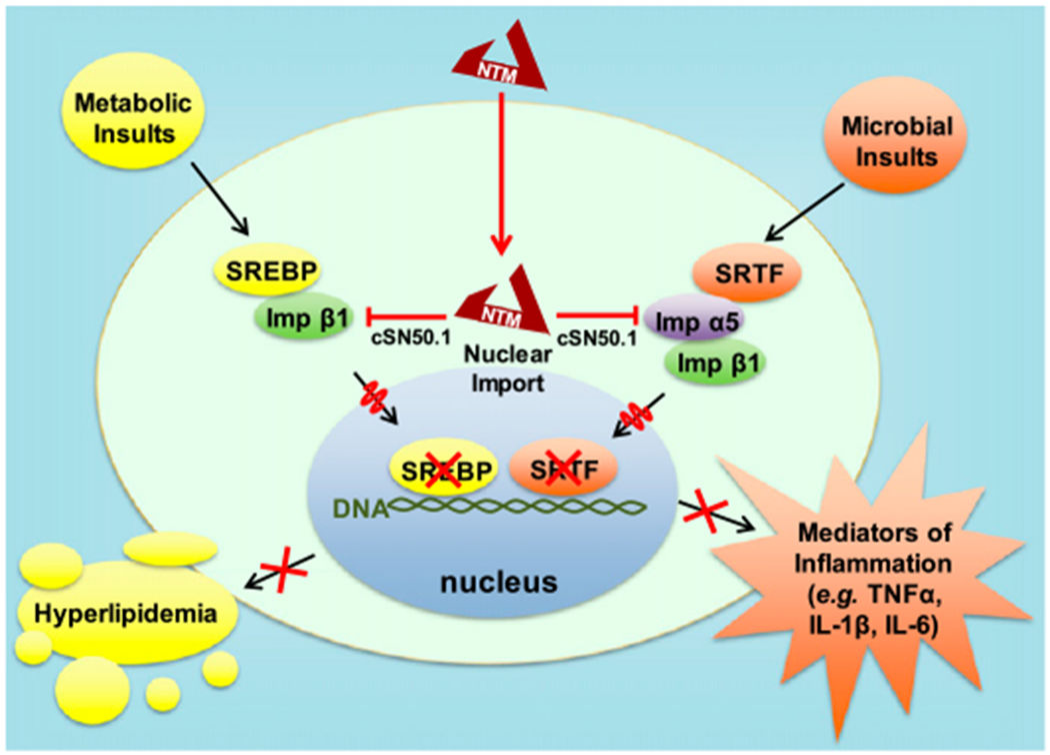
Conceptual depiction of the action of biselective NTM. Biselective NTM simultaneously inhibits nuclear import of stress-responsive transcription factors (SRTFs) mediated jointly by Imp *α*5 and Imp β1, and nuclear translocation of metabolic transcription factors SREBP1 and -2 solely mediated by Imp β1. Biselective NTM suppresses expression of genes that encode mediators of inflammation and proteins participating in synthetic pathways of cholesterol, triglycerides, and fatty acids responsible for hyperlipidemia.

Unexpectedly, we found that cSN50.1 also suppressed sterol regulatory element binding proteins (SREBPs) (9) that govern the expression of genes encoding proteins responsible for the synthetic pathways of cholesterol, triglyceride, and fatty acids (10, 11). We showed that cSN50.1 peptide is a biselective NTM impeding both Imp *α*5–mediated nuclear import of SRTFs and Imp β1–mediated nuclear import of SREBPs (9). Metabolic pathways are deranged in sepsis nonsurvivors (12). LPS also induces an unfolded protein response associated with endoplasmic reticulum stress (2) that activates Caspase 2-mediated processing of the main metabolic transcription factors, SREBPs. Deficiency of SREBP1a provided partial protection from endotoxin shock induced by a relatively low dose of LPS (13). Irrespective of the activation mode of SREBP1 and SREBP2, their common nuclear transport step is mediated solely by Imp β1, targeted by the biselective NTM peptide cSN50.1 that inhibits binding of both Imp *α*5 and Imp β1 to their respective transcription factors (9,14) (Fig. 1). Thus, expression of both proinflammatory genes requiring Imp *α*5–dependent SRTFs and metabolic mediators requiring Imp β1–dependent SREBPs is averted.

The cell-penetrating NTM peptide, cSN50.1, along with its prior congeners (SN50 and cSN50), binds to both Imp *α*5, through an NLS-derived motif, and Imp β1, through a signal-sequence hydrophobic region (SSHR) recognition site (8, 9). Until now, the relative contributions of SRTFs and SREBPs to endotoxin shock could not be determined. We hypothesized that an NTM without the Imp β1–binding junction of cSN50.1 would enable analysis of the impact of SRTFs alone on endotoxin shock survival independent of nuclear transport of SREBPs (9). Therefore, to our knowledge, we developed the first novel selective inhibitor of Imp *α*5–mediated nuclear transport of SRTFs, cSN50.1*α*, and established that nuclear translocation of SRTFs plays the pivotal role in endotoxin shock.

## MATERIALS AND METHODS

### Peptide synthesis and purification

Highly soluble (>100 mg/ml in H_2_O) cell-penetrating NTM peptides, cSN50.1 and cSN50.1*α*, were synthesized as described elsewhere (7–9). These fragment-designed peptides are comprising a cell membrane-translocating motif derived from the SSHR, which also bears the binding site for Imp β1 (9) and an NLS-containing fragment that binds to Imp *α*5 (8). The cSN50.1 sequence is AAVALLPAVLLALLAPCVQRKRQKLMPC, whereas the cSN50.1*α* sequence comprises AAVALLPAVLLA**V**LAPCVQRKRQKLMPC. L to V (bold face) is a loss of function mutation in the Imp β1–binding site, but does not affect the membrane-translocating ability.

### Cell-based assay of nuclear translocation of NF-κB RelA and SREBP2

#### Murine macrophage.

*RAW 264* (RAW) cells (American Type Culture Collection) were cultured according to the manufacturer’s instructions in 10 cm dishes until confluent. Cells were pretreated with 30 μM cSN50.1 or 30 μM cSN50.1*α* 30 min before LPS stimulation (10 ng/ml; *Escherichia coli* O127:B8; Sigma-Aldrich). Cells were incubated for 6 h at 37°C in 5% CO_2_, then harvested, lysed with hypotonic buffer (6) containing 2% NP-40, and washed three times to yield clean nuclei. Nuclear proteins were obtained by high-salt extraction (450 mM NaCl; 4°C, 2000 rpm, 30 min). LPS-stimulated cells not treated with peptide and unstimulated cells served as positive and negative controls, respectively.

#### Human embryonic kidney.

In 10-cm dishes, *293T* (HEK 293T) cells (American Type Culture Collection) were cultured according to the manufacturer’s instructions until confluent. Cells were pretreated for 15 min with 1% hydroxypropyl-β-cyclodextrin (HPCD) in DMEM containing 1% delipidated serum. HPCD was removed, and cells were treated with 30 μM cSN50.1 or cSN50.1*α* in DMEM containing 5% delipidated serum for 2 h (see (9) for details) Nuclear extracts were obtained as described above. HPCD-stimulated cells not treated with peptide and unstimulated cells served as positive and negative controls, respectively.

The nuclear content of NF-κB RelA in RAW cells or SREBP2 in HEK 293T cells was determined by quantitative immunoblotting using rabbit polyclonal anti-NF-κB RelA (Abcam) or rabbit polyclonal anti–SREBP2 (Invitrogen), respectively. Rabbit monoclonal anti-Histone 3 (Cell Signaling Technology) was used to measure Histone 3 as a nuclear loading control for normalization. Immunoblots were analyzed on a LI-COR Biosciences Odyssey Infrared Imaging System.

### Animal study ethics statement

All animal handling and experimental procedures were performed in strict accordance with the recommendations in the Guide for the Care and Use of Laboratory Animals of the National Institutes of Health. The protocol was approved by the Vanderbilt University Animal Care and Use Program (permit number A3227-01). Mice were closely monitored during the course of experiments and euthanized by isoflurane, followed by cervical dislocation when expressed signs of moribund state. Survivors were euthanized at the experimental end point (72 h).

### Endotoxin shock

Two murine models of microbial inflammation were used to determine the nuclear import pathway required for endotoxin shock and ultimate death: 1) high-dose LPS, in which mice were challenged with a lethal dose of LPS (35 mg/kg), and 2) low-dose LPS, in which mice were pretreated with 2-amino-2-deoxy-d-galactosamine (D-Gal) and challenged with nonlethal dose of LPS (50 μg/kg) that was 700 times lower than that employed in the high-dose LPS model. In both experimental models, LPS from *E. coli* strain O127:B8 (Sigma-Aldrich) was used to challenge 8–10-wk-old female C57BL/6 mice (19–20 g body weight; The Jackson Laboratory). Mice were randomized into three groups (five mice per group) and treated i.p. with saline (200 μl/dose) as a control group or cSN50.1 or cSN50.1*α* (both 0.66 mg/dose) peptides reconstituted in 200 μl of water-saline solution (1:1, v/v) as treatment groups.

#### High-dose LPS model.

Mice were challenged with LPS (35 mg/kg, i.p.) and treated by i.p. injection with seven doses of either saline (control group) or NTM peptides (treatment groups) in the following regimen: 30 min before and 30, 90, 150, 210, 360, and 720 min after LPS challenge. Blood samples (~50 μl) were collected from the saphenous vein in EDTA-coated tubes (Sarstedt) before and at 2, 4, 6, 12 and 24 h after LPS challenge.

#### Low-dose LPS model.

Mice were primed by i.p. injection of D-Gal (1 g/kg), causing liver UTP depletion (15). Endotoxin shock was induced by i.p. injection of LPS (50 μg/kg) 45 min after D-Gal administration. Treatment with five doses of saline or NTM peptides was completed as follows: 30 min before and 30, 90, 150, and 210 min after LPS challenge. Blood samples (~50 μl) were collected from the saphenous vein in EDTA-coated tubes (Sarstedt) before and at 90 min and 3 and 6 h after LPS challenge.

### Histology analyses

Organ samples (liver and lung) were collected and fixed overnight in 10% formalin, routinely processed, embedded in paraffin, sectioned at 5 μm, and stained with H&E or Periodic acid–Schiff/hematoxylin to assess injury, hemorrhage, and liver glycogen stores, respectively. Immunohistochemistry analyses with Abs against VCAM-1 (Abcam), neutrophils (Abcam), and Caspase-3 (Cell Signaling Technology) were performed on the Leica BOND-MAX following standard protocols in the Translational Pathology Shared Resource at Vanderbilt University Medical Center.

### Cytokine/chemokine assays

A cytometric bead array (BD Biosciences) was used to measure TNF-*α*, IL-6, IFN-*γ*, and MCP-1 in murine blood plasma following the manufacturer’s protocol and analyzed in the Vanderbilt Medical Center Flow Cytometry Shared Resource as previously described (7).

### Statistical analysis

Prism 6 software (GraphPad) was used for statistical analyses. Cytokine and chemokine levels in plasma collected from the same animals at different time points were evaluated by repeated measures two-way ANOVA with Sidak posttest. Survival data were plotted as Kaplan–Meier survival curves and analyzed by the log-rank test. Immunoblots of nuclear extracts were analyzed by ordinary one-way ANOVA with Holm–Sidak test for multiple comparison. Data are presented as the mean ± SEM. The *p* values <0.05 were considered significant.

## RESULTS

### The cSN50.1α peptide impedes nuclear translocation of proinflammatory transcription factor NF-κB RelA while sparing nuclear transport of metabolic transcription factor SREBP2

**FIGURE 2.**
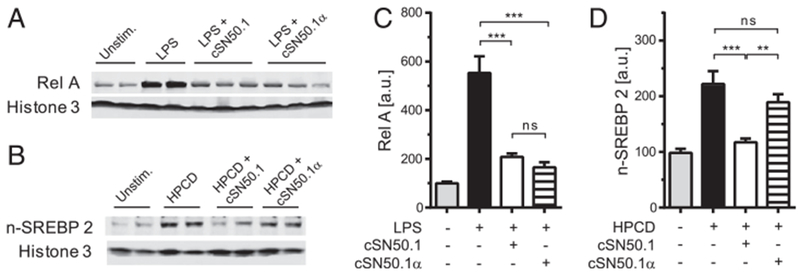
Imp *α*5–selective NTM impedes nuclear translocation of proinflammatory transcription factor NF-κB RelA while sparing Imp β1–mediated nuclear translocation of metabolic transcription factor SREBP2. (**A**) Immunoblot analysis of nuclear NF-κB RelA in RAW 264 cells. Cells were treated with 30 μM NTM peptides as indicated for 30 min then stimulated with 10 ng/ml LPS for 6 h. (**B**) Immunoblot analysis of nuclear SREBP2 in HEK 293T cells. Cells were depleted of lipids by treatment with HPCD and treated with 30 μM NTM peptides as indicated for 2 h. (**C** and **D**) Quantitative representation of immunoblots shown in (A) and (B), respectively. Data presented in this figure represent two independent in vitro experiments completed in duplicates or triplicates (as indicated). All signals were normalized to Histone 3 and expressed as percent inhibition ± SEM. Significance was determined by one-way ANOVA. **p < 0.005, ***p < 0.005.

Imp *α* recognize and bind their cargo, (i.e., transcription factors) for nuclear delivery. In turn, the primary junction of Imp β1 is to bind cargo-carrying Imp *α* and ferry the supramolecular complex through the nuclear pores (7–9). A second function of Imp β1, independent of Imp *α*, mediates nuclear import of SREBPs, the master regulators of metabolic pathways (Fig. 1) (14). Thus, NLS-based nuclear import of SRTFs is mediated by Imp *α*5 (8), whereas nuclear import of SREBP1 and SREBP2 is mediated by Imp β1 (9). Therefore, we developed an NTM that solely targets Imp *α*5–mediated import to determine the role of SRTFs in endotoxin shock. The cell-penetrating cSN50.1*α* peptide has an intact NLS fragment responsible for interaction with Imp *α*5 but contains a mutated SSHR fragment, disabling its binding to Imp β1 that transports SREBPs to the nucleus (8, 9) while retaining the ability to cross the cell membrane. We compared the inhibitory effect of this new, novel peptide with that of the biselective cSN50.1 peptide on Imp *α*5–mediated nuclear translocation of NF-κB RelA and Imp β1–mediated nuclear translocation of SREBPs, which regulate genes responsible for lipogenesis. The biselective cSN50.1 peptide inhibited Imp *α*5–mediated nuclear translocation of NF-κB RelA in LPS-stimulated murine macrophage cells (RAW 246) as well as Imp β1–mediated nuclear transport of SREBP2 in lipid-depleted human embryonic kidney cells (HEK 297T) (Fig. 2). In contrast, the new monoselective cSN50.1*α* peptide inhibited only Imp *α*5–mediated nuclear transport of NF-κB RelA while sparing Imp β1–mediated nuclear transport of SREBP2. Hence, we established the selective action of cSN50.1*α* toward Imp *α*5–mediated import of NF-κB RelA and other SRTFs.

### Which nuclear import pathway is required for death from endotoxin shock?

After establishing selectivity of the new cSN50.1*α* peptide in cell-based assays, we analyzed the novel Imp *α*5–selective peptide in two well-known models of endotoxin shock.

**FIGURE 3.**
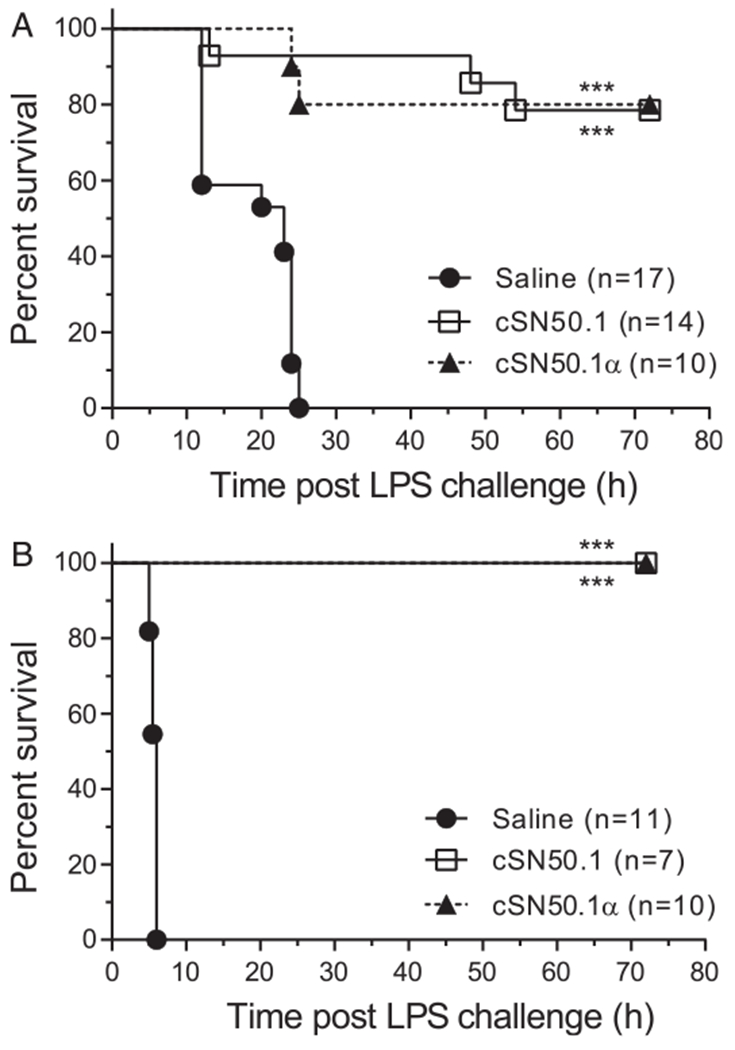
Imp *α*5–selective NTM (cSN50.1*α*) increases survival in endotoxin shock in two models. (**A**) High-dose LPS model. Mice were challenged with a high dose of LPS (35 mg/kg). (**B**) Low-dose LPS model. Mice were primed with d-galactosamine (1 g/kg) and challenged with a nonlethal amount of LPS (50 μg/kg). Mice were treated with either seven (A) or five (B) doses of NTM (0.66 mg/dose) or saline. Data presented in this figure denote at least two independent in vivo experiments. Number of mice per condition group is indicated on the graph. Kaplan–Meier survival plot with *p* value calculated by log-rank analysis. ***p < 0.0005.

In the first model, based on challenge with a high dose of LPS (35 mg/kg), all saline-treated control mice died because of endotoxin shock within 24 h (Fig. 3A). Strikingly, 80% of mice treated with the new Imp *α*5–selective peptide survived for at least 72 h. Subsequently, we extended the observation time to 7 d with a similar outcome (data not shown). In agreement with a previous study (7), 80% survival was also observed in mice treated with the biselective peptide, cSN50.1, which simultaneously targets both Imp *α*5 and Imp β1. Thus, the Imp β1–mediated pathway, which transports SREBPs, does not contribute significantly to mortality from endotoxin shock in this model.

In a second hyperacute endotoxin shock model, D-Gal-primed mice were challenged with a low dose of LPS (50 μg/kg) (15,16). However, D-Gal not only sensitizes liver cells to the proapoptotic activity of LPS-induced TNF-*α*, but also increases TNF-*α* expression (17). Rapid death of saline-treated control mice ensued within 6 h. In striking contrast, all mice treated with the new Imp *α*5–selective peptide survived at least 72 h, similarly to mice treated with the biselective peptide (Fig. 3B). Thus, the Imp *α*5–mediated transport of SRTFs contributes to lethality, whereas the Imp β1–mediated transport of SREBPs is not essential in both models of endotoxin shock.

### Selective targeting of Imp α5–mediated transport of SRTFs by the cSN50.1α peptide suppresses inflammatory mediators and organ injury

**FIGURE 4.**
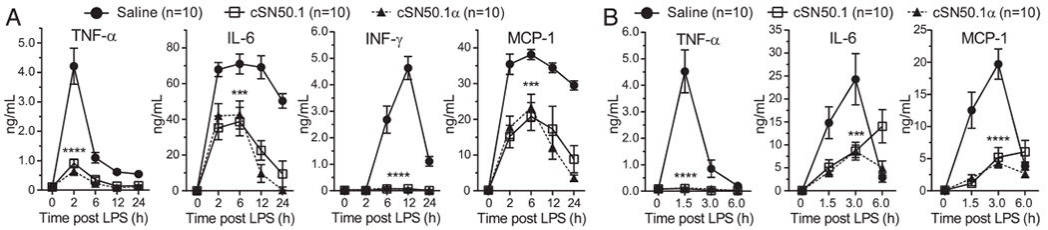
Imp *α*5–selective NTM, cSN50.1*α* prevents cytokine/chemokine expression in endotoxin shock. Blood plasma levels of cytokines, TNF-*α*, IL-6, INF-*γ*, and chemokine MCP-1 in a high-dose LPS model (35 mg/kg LPS) (**A**) and a low-dose LPS model (1 g/kg D-Gal then 50 μg/kg LPS) (**B**). Mice were treated with either seven (A) or five (B) doses of NTM (0.66 mg/dose) or saline. Data presented in this figure denote two independent in vivo experiments completed with five mice per condition group. Data are expressed as a mean ± SEM (*n* = 10 mice per group; *p* values calculated by two-way repeated measures ANOVA). ***p < 0.0005, ****p < 0.0001.

**FIGURE 5.**
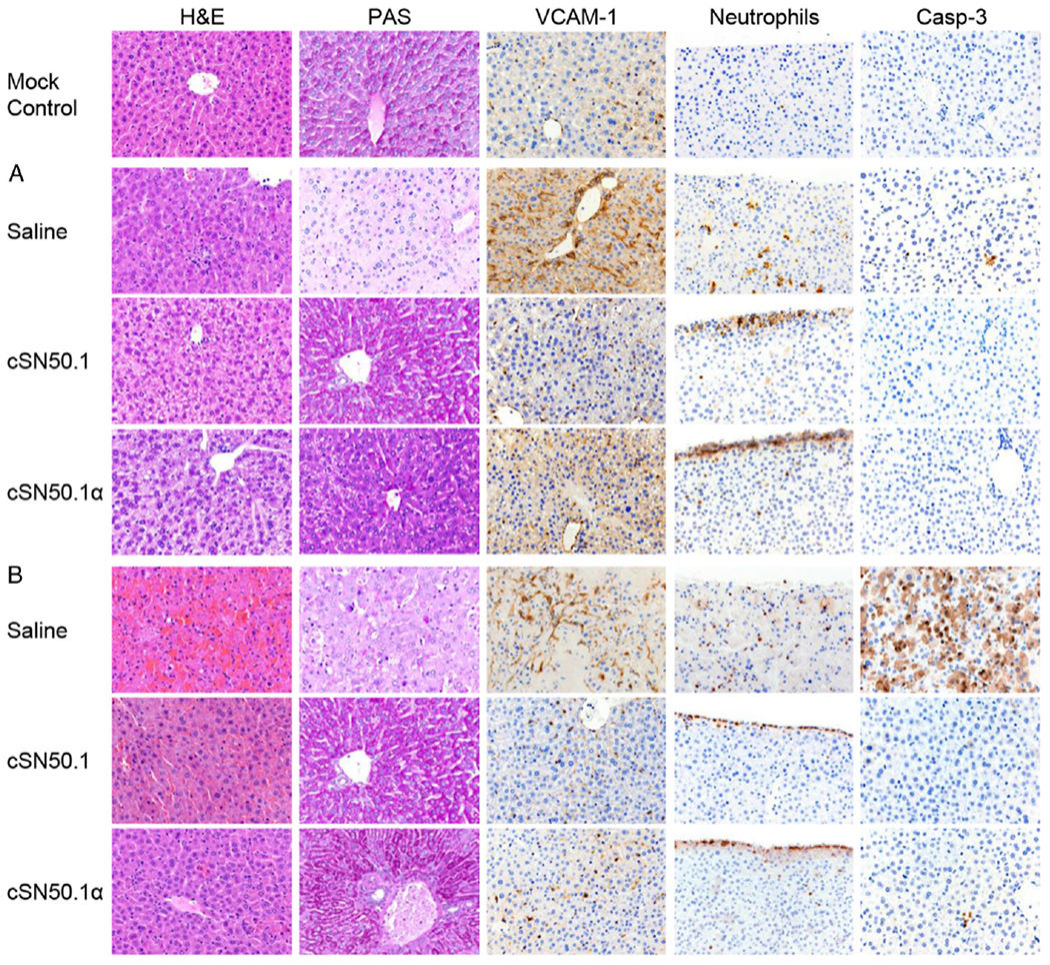
Imp *α*5–selective NTM protects liver from LPS-induced glycogenolysis (PAS), endothelial injury (VCAM-1), and neutrophil infiltration in a high-dose LPS model and from massive apoptosis (Caspase 3) in a low-dose LPS model of endotoxin shock. Representative images (original magnification ×40) of liver sections in unchallenged mice (mock control) or mice challenged with LPS. Data presented in this figure denote two independent in vivo experiments completed with five mice per condition group [(**A**) 35 mg/kg LPS; (**B**) 1 g/kg D-Gal then 50 μg/kg LPS] and treated with saline or cSN50.1 or cSN50.1*α* peptides (both at 0.66 mg/dose). See Materials and Methods for details.

Consistent with the survival results, the cSN50.1*α* peptide suppressed plasma levels of proinflammatory cytokines (TNF-*α*, IL-6, and INF-*γ*) and chemokine MCP-1. The kinetics of these key mediators’ expression were similarly reduced in animals treated with the biselective cSN50.1 peptide (7) (Fig. 4A). The dramatically faster time course of inflammatory mediators, cytokines, and a chemokine was recorded in the low-dose LPS plus D-Gal model, whereas the peak values were similar (Fig. 4B). In this model, D-Gal, a metabolic inhibitor, precipitously depletes UTP in hepatocytes, thereby predisposing them to massive apoptosis (15). Indeed, massive apoptosis of the liver cells reflected by the activation of Caspase 3 was prominently displayed in untreated mice, whereas it was barely detectable in the high-dose LPS model. Strikingly, apoptosis was suppressed by the cSN50.1*α* peptide (Fig. 5B).

Endotoxin stimulates glycogenolysis in the liver (18). Rapid or chronic depletion of hepatic glycogen is detrimental in bacterial and viral infections (19). Prevention of glycogenolysis by the Imp *α*5–selective peptide, as well as by the biselective cSN50.1 peptide, is associated with the survival of mice in both models of endotoxin shock (see PAS staining Fig. 5A) and D-Gal–primed mice challenged with a low dose of LPS (Fig. 5B). Thus, Imp *α*5–selective peptide, cSN50.1*α*, is sufficient to protect liver from LPS-induced metabolic derangements, especially in the D-Gal–primed mice model known for a fulminant course of endotoxin shock (16).

As the liver’s sinusoids are lined with sinusoidal endothelial cells, stellate cells, and Kupffer cells (20), circulating LPS interacts with its cognate receptor, TLR4, expressed on these strategic mainstays of liver injury. LPS-induced endothelial injury is manifested by increased expression of VCAM-1 and increased neutrophil trafficking into the liver parenchyma, leading to increased apoptosis as documented by Caspase 3 activation (Fig. 5). These hallmarks of inflammatory liver injury were abrogated in mice treated with Imp *α*5–selective peptide that stopped LPS-induced neutrophil trafficking at the liver’s edge, thereby preventing their infiltration into the liver’s parenchyma. Inflammatory changes were also suppressed by Imp *α*5–selective peptide in the lungs (Supplemental Fig. 1).

### The cSN50.1α peptide impedes nuclear translocation of proinflammatory stress-responsive transcription factors NF-κB RelA and STAT1 in the liver of mice challenged with LPS

Imp *α*5–selective peptide suppressed nuclear translocation of transcription factor NF-κB RelA and STAT1, similar to the suppression by biselective peptide (cSN50.1). Thus, the nuclear transport of Imp *α*5–ferried SRTFs, which are required for the expression of inflammatory mediators, plays a key role in endotoxin shock (Supplemental Fig. 2).

## DISCUSSION

Taken together, our results identify a new mechanism of endotoxin shock that depends on Imp *α*5–mediated nuclear transport of SRTFs rather than Imp β1–mediated nuclear translocation of SREBPs. Therefore, the SREBP-regulated genes that encode proteins involved in the homeostasis of cholesterol, triglycerides, and fatty acids are not primary contributors to lethality in endotoxin shock. Although previous studies reported that SREBP1a-deficient mice are resistant to endotoxin shock (13), the dose of LPS was unusually low, and the death-averting effect was only partial. The impact of the inborn deficiency of the SREBP1a gene on development of the innate immune system and its responsiveness to LPS requires consideration. For example, SREBP2 also *trans*-regulates Notch pathway genes required for hematopoiesis (21). Hence, this hematopoietic pathway is also likely to be spared by the cSN50.1*α* peptide.

The utility of a novel cell-penetrating peptide that selectively suppresses Imp *α*5–mediated nuclear transport of SRTFs while allowing SREBPs access to the nucleus is multifaceted. It offers a hitherto unavailable approach to the differential analysis of distinct pathways of nuclear signaling mediated by transcription factors. This approach can be extended to the mechanistic analysis of microbial inflammation caused by other agents as well as inflammation due to allergic, autoimmune, metabolic, and physical insults (2).

In summary, the novel approach to microbial inflammation reported in this study offers delineation of signaling pathways to the nucleus of immune and nonimmune cells. The essential pathway depends on Imp *α*5–mediated nuclear translocation of SRTFs.

## Supplementary Material

Suppl Inf.

## Abbreviations used in this article:

D-Gal: 2-amino-2-deoxy-d-galactosamine
HPCD: hydroxypropyl-β-cyclodextrin
Imp *α*: importin *α*
NLS: nuclear localization sequence
NTM: nuclear transport modifier
SREBP: sterol regulatory element binding protein
SSHR: signal-sequence hydrophobic region

## References

[1] Andreasen AS, Krabbe KS, Krogh-Madsen R, Taudorf S, Pedersen BK, and Møller K. 2008 Human endotoxemia as a model of systemic inflammation. Curr. Med. Chem 15: 1697–1705. 18673219 10.2174/092986708784872393

[2] Hawiger J, and Zienkiewicz J. 2019 Decoding inflammation, its causes, genomic responses, and emerging countermeasures. Scand. J. Immunol DOI: 10.1111/sji.12812. PMC6883124 31378956

[3] Beutler BA 2009 TLRs and innate immunity. Blood 113: 1399–1407. 18757776 10.1182/blood-2008-07-019307 PMC2644070

[4] Opal SM 2010 New perspectives on immunomodulatory therapy for bacteraemia and sepsis. Int. J. Antimicrob. Agents 36(Suppl. 2): S70–S73. 10.1016/j.ijantimicag.2010.11.008 21129935

[5] Hawiger J 2001 Innate immunity and inflammation: a transcriptional paradigm. Immunol. Res 23: 99–109. 11444396 10.1385/IR:23:2-3:099

[6] Veach RA, Liu Y, Zienkiewicz J, Wylezinski LS, Boyd KL, Wynn JL, and Hawiger J. 2017 Survival, bacterial clearance and thrombocytopenia are improved in polymicrobial sepsis by targeting nuclear transport shuttles. PLoS One 12: e0179468. 28628637 10.1371/journal.pone.0179468 PMC5476269

[7] DiGiandomenico A, Veach RA, Zienkiewicz J, Moore DJ, Wylezinski LS, Hutchens MA, and Hawiger J. 2014 The “genomic storm” induced by bacterial endotoxin is calmed by a nuclear transport modifier that attenuates localized and systemic inflammation. PLoS One 9: e110183. 25329889 10.1371/journal.pone.0110183 PMC4203769

[8] Zienkiewicz J, Armitage A, and Hawiger J. 2013 Targeting nuclear import shuttles, importins/karyopherins alpha by a peptide mimicking the NFκB1/p50 nuclear localization sequence. J. Am. Heart Assoc 2: e000386. 24042087 10.1161/JAHA.113.000386 PMC3835248

[9] Liu Y, Major AS, Zienkiewicz J, Gabriel CL, Veach RA, Moore DJ, Collins RD, and Hawiger J. 2013 Nuclear transport modulation reduces hypercholesterolemia, atherosclerosis, and fatty liver. J. Am. Heart Assoc 2: e000093. 23563994 10.1161/JAHA.113.000093 PMC3647260

[10] Kim JY, Garcia-Carbonell R, Yamachika S, Zhao P, Dhar D, Loomba R, Kaufman RJ, Saltiel AR, and Karin M. 2018 ER stress drives lipogenesis and steatohepatitis via caspase-2 activation of S1P. Cell 175: 133–145.e15. 30220454 10.1016/j.cell.2018.08.020 PMC6159928

[11] Horton JD, Goldstein JL, and Brown MS. 2002 SREBPs: activators of the complete program of cholesterol and fatty acid synthesis in the liver. J. Clin. Invest 109: 1125–1131. 11994399 10.1172/JCI15593 PMC150968

[12] Hawiger J, Veach RA, and Zienkiewicz J. 2015 New paradigms in sepsis: from prevention to protection of failing microcirculation. J. Thromb. Haemost 13: 1743–1756. 26190521 10.1111/jth.13061 PMC5014149

[13] Im SS, Yousef L, Blaschitz C, Liu JZ, Edwards RA, Young SG, Raffatellu M, and Osborne TF. 2011 Linking lipid metabolism to the innate immune response in macrophages through sterol regulatory element binding protein-1a. Cell Metab. 13: 540–549. 21531336 10.1016/j.cmet.2011.04.001 PMC3090630

[14] Lee SJ, Sekimoto T, Yamashita E, Nagoshi E, Nakagawa A, Imamoto N, Yoshimura M, Sakai H, Chong KT, Tsukihara T, and Yoneda Y. 2003 The structure of importin-beta bound to SREBP-2: nuclear import of a transcription factor. Science 302: 1571–1575. 14645851 10.1126/science.1088372

[15] Galanos C, Freudenberg MA, and Reutter W. 1979 Galactosamine-induced sensitization to the lethal effects of endotoxin. Proc. Natl. Acad. Sci. USA 76: 5939–5943. 293694 10.1073/pnas.76.11.5939 PMC411768

[16] Liu D, Li C, Chen Y, Burnett C, Liu XY, Downs S, Collins RD, and Hawiger J. 2004 Nuclear import of proinflammatory transcription factors is required for massive liver apoptosis induced by bacterial lipopolysaccharide. J. Biol. Chem 279: 48434–48442. 15345713 10.1074/jbc.M407190200

[17] Endo Y, Shibazaki M, Yamaguchi K, Kai K, Sugawara S, Takada H, Kikuchi H, and Kumagai K. 1999 Enhancement by galactosamine of lipopolysaccharide(LPS)-induced tumour necrosis factor production and lethality: its suppression by LPS pretreatment. Br. J. Pharmacol 128: 5–12. 10498828 10.1038/sj.bjp.0702747 PMC1571593

[18] Casteleijn E, Kuiper J, Van Rooij HC, Kamps JA, Koster JF, and Van Berkel TJ. 1988 Endotoxin stimulates glycogenolysis in the liver by means of intercellular communication. J. Biol. Chem 263: 6953–6955. 3284878

[19] Sakr SA, Badrah GA, and Sheir RA. 2013 Histological and histochemical alterations in liver of chronic hepatitis C patients with *Helicobacter pylori* infection. Biomed. Pharmacother 67: 367–374. 23659901 10.1016/j.biopha.2013.03.004

[20] Kmiec Z 2001 Cooperation of liver cells in health and disease In Advances in Anatomy, Embryology and Cell Biology, Vol. 161 Springer, New York, p. 13–28. 10.1007/978-3-642-56553-3 11729749

[21] Gu Q, Yang X, Lv J, Zhang J, Xia B, Kim JD, Wang R, Xiong F, Meng S, Clements TP, 2019 AIBP-mediated cholesterol efflux instructs hematopoietic stem and progenitor cell fate. Science 363: 1085–1088. 30705153 10.1126/science.aav1749 PMC6469354

