# Supplementary material for: Protection from Endotoxin Shock by Selective Targeting of Proinflammatory Signaling to the Nucleus Mediated by Importin Alpha 5
Source: Immunohorizons. Author manuscript; Available in PMC 2019 Sep 30. (PMC6768080; doi:10.4049/immunohorizons.1900064)
Supplement: Suppl Inf. [file NIHMS1051329-supplement-Suppl_Inf_.pdf]

Supplemental Materials.

Supplemental Figure 1

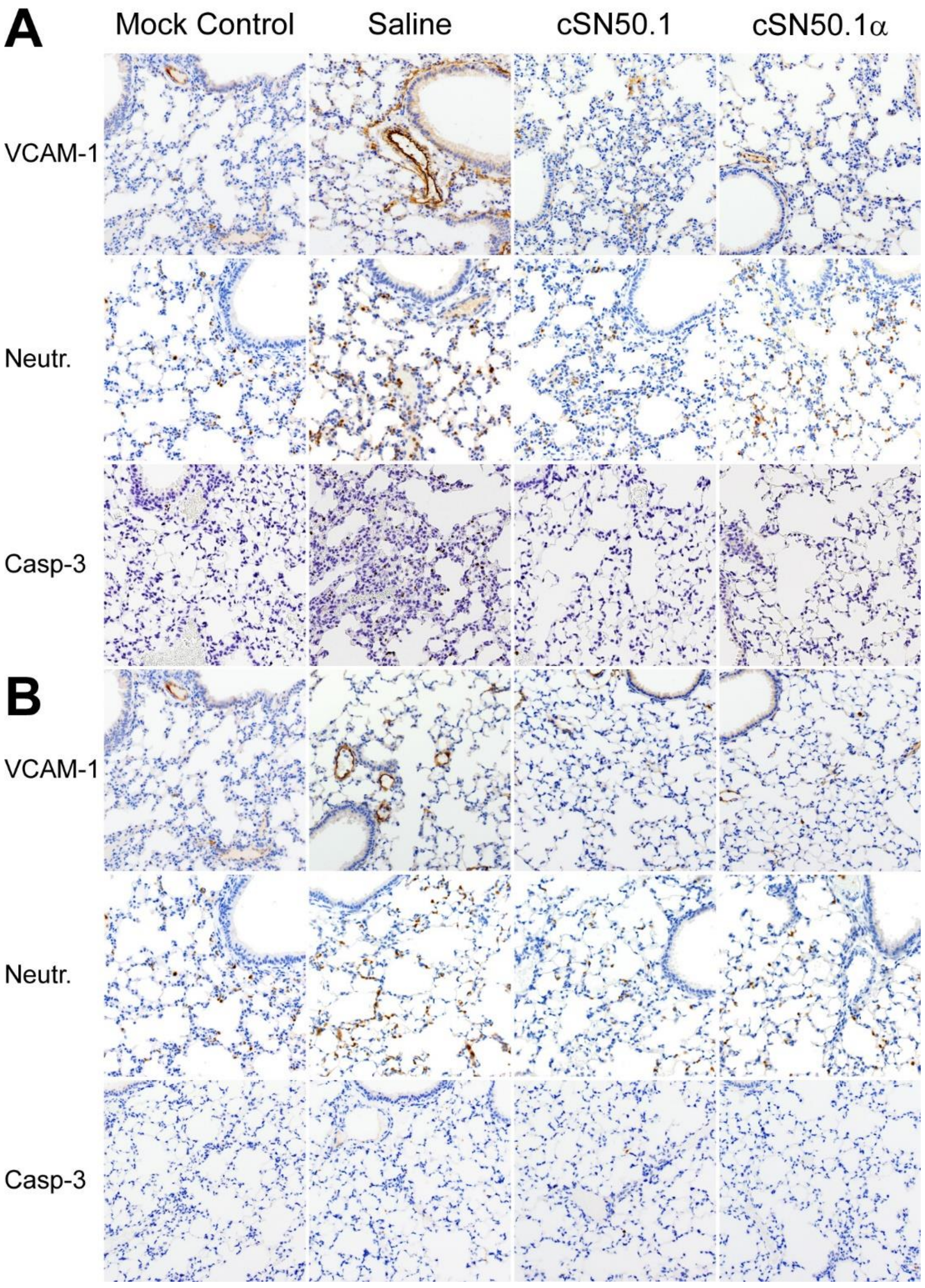

Supplemental Fig. 1. Importin α5-selective NTM suppresses lung endothelial injury (VCAM-1), decreases circulating neutrophils, and prevents apoptosis (Casp-3) in mice comprising 2 models of Endotoxin Shock: High Dose LPS model (A) and Low Dose LPS model primed with D-galactosamine (B). Representative images (40x magnification) of lung sections in unchallenged mice (mock control) or mice challenged with LPS (**A:** 35 mg/kg LPS; **B:** 1 g/kg D-Gal then 50 µg/kg LPS). Mice were treated with either 7 (A) or 5 (B) doses of NTM (33 mg/kg) or saline (See text for details). Data presented in this figure denotes 2 independent in vivo experiments completed with 5 mice per condition group.

Supplemental Figure. 2

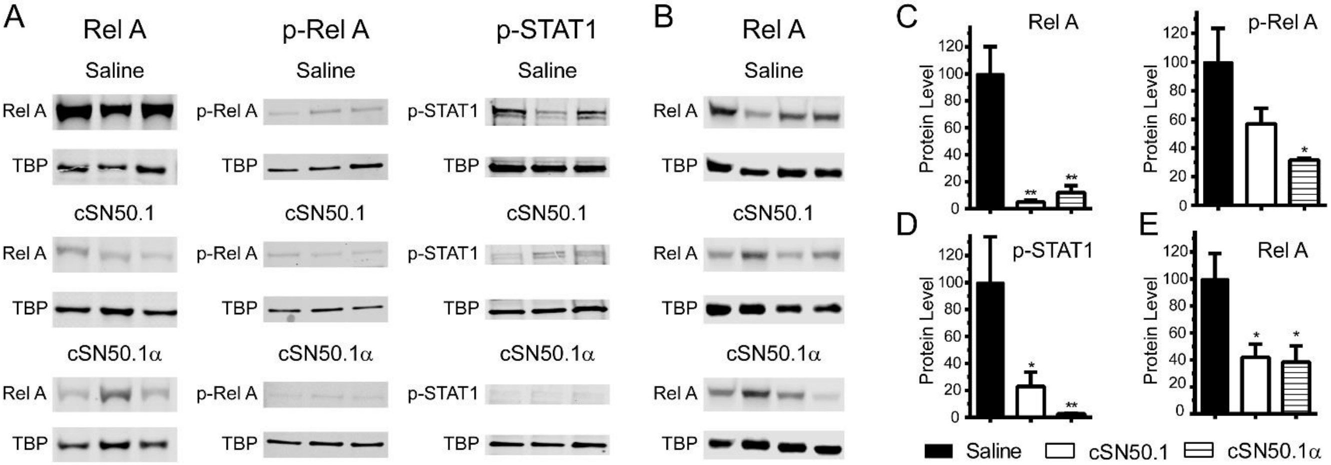

Supplemental Fig. 2. Importin α5-selective NTM reduces nuclear translocation of NF-κB p65 (Rel A), phospho-NF-κB p65 (p-Rel A) and phospho-STAT1 (α and β) in two models of Endotoxin Shock. A. and **B.** Representative immunoblots of liver nuclear extracts. Liver samples were collected 12 hr after mice were challenged with a High Dose LPS (**A**) or 6 hr after mice primed with D-Gal were challenged with a Low Dose LPS (**B**). Mice were treated with either 7 (A) or 5 (B) doses of NTM (33 mg/kg) or saline. **C.** and **D.** Quantitative representation of immunoblots shown in panel **A**. **E.** Quantitative representation of immunoblots shown in panel **B**. Data presented in this figure denotes 2 independent *in vivo* experiments completed with 5 mice per condition group. All signals were normalized to TATA Binding Protein (TBP) and expressed as percent inhibition ± SEM. Significance was determined by one-way ANOVA, * - p<0.05, ** - p<0.005.
